# The Cornell COVID-19 Testing Laboratory: A Model to High-Capacity Testing Hubs for Infectious Disease Emergency Response and Preparedness

**DOI:** 10.3390/v15071555

**Published:** 2023-07-15

**Authors:** Melissa Laverack, Rebecca L. Tallmadge, Roopa Venugopalan, Daniel Sheehan, Scott Ross, Rahim Rustamov, Casey Frederici, Kim S. Potter, François Elvinger, Lorin D. Warnick, Gary A. Koretzky, Robert Lawlis, Elizabeth Plocharczyk, Diego G. Diel

**Affiliations:** 1 Department of Population Medicine and Diagnostic Sciences, Animal Health Diagnostic Center (AHDC), College of Veterinary Medicine, Cornell COVID-19 Testing Laboratory (CCTL), Cornell University, Ithaca, NY 14853, USArlt8@cornell.edu (R.L.T.);; 2 Information Technology, College of Veterinary Medicine, Cornell University, Ithaca, NY 14853, USA; 3 Cayuga Medical Center, Cayuga Health System, Ithaca, NY 14850, USA; 4 Department of Microbiology and Immunology, College of Veterinary Medicine, Cornell University, Ithaca, NY 14853, USA; 5 Department of Medicine, Weill Cornell Medicine, Cornell University, New York City, NY 10065, USA

**Keywords:** COVID-19, SARS-CoV-2, diagnostic workflow, surveillance

## Abstract

The unprecedented COVID-19 pandemic posed major challenges to local, regional, and global economies and health systems, and fast clinical diagnostic workflows were urgently needed to contain the spread of SARS-CoV-2. Here, we describe the platform and workflow established at the Cornell COVID-19 Testing Laboratory (CCTL) for the high-throughput testing of clinical samples from the university and the surrounding community. This workflow enabled efficient and rapid detection and the successful control of SARS-CoV-2 infection on campus and its surrounding communities. Our cost-effective and fully automated workflow enabled the testing of over 8000 pooled samples per day and provided results for over 2 million samples. The automation of time- and effort-intensive sample processing steps such as accessioning and pooling increased laboratory efficiency. Customized software applications were developed to track and store samples, deconvolute positive pools, track and report results, and for workflow integration from sample receipt to result reporting. Additionally, quality control dashboards and turnaround-time tracking applications were built to monitor assay and laboratory performance. As infectious disease outbreaks pose a constant threat to both human and animal health, the highly effective workflow implemented at CCTL could be modeled to establish regional high-capacity testing hubs for infectious disease preparedness and emergency response.

## 1. Introduction

The novel coronavirus now known as severe acute respiratory syndrome coronavirus 2 (SARS-CoV-2) emerged in China at the end of 2019 and spread worldwide, causing an unprecedented pandemic [1,2,3]. Infection with SARS-CoV-2 was particularly difficult to control because nearly half of the individuals infected do not develop symptoms [4], yet still spread SARS-CoV-2 to others for several days post-infection [4]. Additionally, individuals that experience symptoms caused by SARS-CoV-2 infection can spread the virus up to 5 days prior to becoming symptomatic, being responsible for over 40% of secondary cases [5,6]. Infectious virus is detected for up to 5–8 days after the onset of symptoms [7,8,9,10]. Thus, control of SARS-CoV-2 transmission is difficult, particularly in densely populated settings [11,12,13]. Surveillance testing and the early detection of positive cases are therefore essential to curb virus spread and control the number of cases and infections.

University settings offer many opportunities for interactions of groups of individuals that originate from across the globe. University campuses offer many shared spaces designed for a variety of academic, dining, and residence purposes and experiences. Additionally, social gatherings are abundant. These features complicate the control of SARS-CoV-2 transmission on a university campus. Recognizing the characteristics of viral transmission and the unique environment of the university campus, a surveillance testing program was designed at Cornell University during the summer of 2020. A key aspect of this program was the frequent testing of the entire university community including asymptomatic individuals for SARS-CoV-2. We reasoned that on-campus SARS-CoV-2 testing would enable the university to (1) rapidly identify individuals infected with SARS-CoV-2 so they could be isolated and/or obtain prompt medical attention; (2) perform contact tracing to rapidly identify potential pre-symptomatic or asymptomatic secondary cases and limit further spread; (3) provide SARS-CoV-2 incidence case counts and the prevalence of positive samples for the campus community and local public health authorities; and (4) reveal the dynamics of SARS-CoV-2 transmission within the university community.

As a central pillar of the Cornell surveillance program, we established a high-throughput SARS-CoV-2 testing laboratory, the Cornell COVID-19 Testing Laboratory (CCTL). The objective of the CCTL was to offer pooled testing with minimal result turnaround times to enable the rapid isolation of infected individuals and quarantine contacts. To meet that objective, our workflow needed to accommodate the testing of pooled or individual samples simultaneously. Surveillance samples were pooled before testing to maximize the testing capacity and decrease the cost of testing. Individual testing was included in the laboratory workflow to deconvolute SARS-CoV-2-positive pools and identify positive individuals, to test specimens for contact tracing efforts, to test specimens collected from symptomatic individuals, or for testing when the probability of a sample being positive was high enough to limit the advantage of pooling. The Cornell COVID-19 surveillance schedule and other public health measures taken have been described elsewhere [14].

Given the anticipated high-volume testing needs (8000 samples per day), we identified the steps in the workflow that were the most time- and labor-intensive and implemented automation. Accessioning thousands of specimens per day is a time-consuming step. Pooling specimens is both time- and labor-intensive. Manually labeling runs with specimen identifiers is another time-intensive step where errors could be introduced. Manual sample and reagent transfer between 96-well plates are repetitive steps with ergonomic stressors. All these steps were targeted for automation using either robotics or electronic data transfer methods.

The premise of high-throughput pooled testing posed significant information technology (IT) challenges. Once the testing strategy and testing workflow were designed, our IT team developed efficient software applications to support the workflow without the need for users to switch between different applications or to access the laboratory information management system (LIMS). Customized application software and scripts were essential to minimize hands-on input and achieve a streamlined automated workflow.

Another important consideration was the selection of the SARS-CoV-2 assay to be used at CCTL. An RT-PCR that offered high sensitivity to allow pooling and high accuracy to limit the number of repeated tests was optimal. To reduce the risk of delays due to a potential testing reagent shortage, two RT-PCR tests were selected for the initial evaluation and validation of the workflow: (1) the EZ-SARS-CoV-2 assay developed by Tetracore Inc. (Rockville, MD, USA) and (2) the TaqPath COVID-19 Combo Kit Multiplex Real-Time RT-PCR assay (Thermo Fisher Scientific Inc., Waltham, MA, USA) [15] which had Emergency Use Authorization from the United States Food and Drug Administration, with the former being selected [15] and implemented for testing at CCTL.

Considering the short time frame available to launch the laboratory, the workflow incorporated experienced faculty and staff from the Animal Health Diagnostic Center (AHDC)—a Level 1 member of the National Animal Health Laboratory Network (NAHLN)—and PCR instrumentation routinely used at AHDC for the testing of animal clinical samples. Similarly, the IT staff and application developers were familiar with the needs specific to diagnostic testing. CCTL was operated in partnership with Cayuga Medical Center (CMC) as they provided critical expertise in the regulations governing human diagnostic testing, quality control, and human laboratory testing operations. Herein, we describe the workflow that was designed and implemented to meet the considerations above and to optimize the laboratory’s performance.

## 2. Materials and Methods

### 2.1. Laboratory Workflow Overview

**Figure 1:**
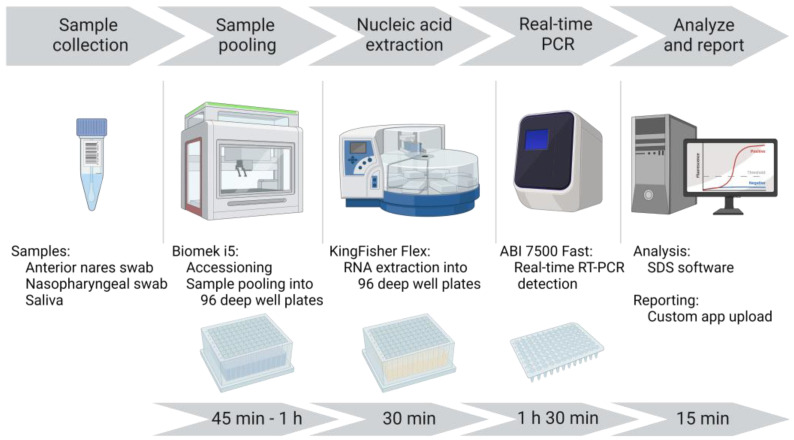
CCTL workflow overview. Samples (anterior nares swabs, nasopharyngeal swabs, or saliva) were collected into 5 mL tubes labeled with unique barcode identifiers. Tubes were loaded onto the Biomek i5 liquid handler which read the barcodes on tubes and racks and pooled the samples into 96-deep-well plates. Nucleic acid was extracted using the Applied Biosystems KingFisher Flex Magnetic Particle Processor. RT-PCR amplification and detection occurred on the ABI 7500 Fast platform. After analysis, the results were uploaded to a custom app that performed quality control checks and interpreted results. The approximate duration of each step is shown. Figure created with BioRender.com, last accessed 5 June 2023.

A unidirectional workflow was designed for high-throughput SARS-CoV-2 testing to support Cornell University’s SARS-CoV-2 surveillance program. Five rooms in a BSL-2 laboratory space were designated to separate and isolate specific steps in the testing process: (1) specimen receiving, processing, and pooling; (2) nucleic acid extraction; (3) RT-PCR mastermix preparation; (4) RT-PCR setup; and (5) a large room with discrete benches dedicated for extraction reagent preparation, real-time PCR instruments, and a computer area for app usage and result analysis. In brief, the workflow began with specimen receipt and proceeded to automated accessioning into LIMS and sample transfer and pooling using Biomek i5 liquid handler automated workstations (Figure 1). Sample plates were loaded into Kingfisher Flex instruments for automated nucleic acid extraction. SARS-CoV-2 amplification and detection were performed using ABI 7500 Fast real-time PCR instruments. After the analysis of the results, SARS-CoV-2-positive pools were deconvoluted to identify individual positive specimens.

### 2.2. Specimen Collection

Our previous study determined that anterior nares (AN) swab specimens were appropriate for SARS-CoV-2 diagnostic testing, whether obtained from symptomatic individuals or asymptomatic individuals for surveillance purposes [10]. Multiple specimen collection sites were established across the campus where Cornell University community members could self-collect AN swabs and place them in 5 mL tubes pre-filled with 1 mL of viral transport media (VTM, Corning Transport Medium). Barcoded specimen identification labels were printed via Brady i7100 printers using Brady Workstation software version 4.9.1.2 (Brady Corporation, Milwaukee, WI, USA) during the sample collection process and applied to the tube. The specimen label design and high print quality were critical for the barcode scanners to read the identification correctly. Specimens were transported to CCTL from on-campus collection sites or CMC by couriers. Nasopharyngeal (NP) swab specimens were collected by health care providers and placed in tubes pre-filled with 1 mL VTM, submitted to Cayuga Medical Center, and delivered to CCTL. Saliva specimens were self-collected in 5 mL tubes, submitted to Cayuga Medical Center if off-campus, and delivered to CCTL.

### 2.3. Specimen Processing

Specimens were delivered to the CCTL in 5 mL conical tubes and identified with unique barcode identification labels. Staff wore enhanced PPE exceeding the requirements for BSL-2 when inspecting, processing, and transferring specimens. Specimens were inspected to confirm that each tube contained either one swab with VTM or saliva, with a single legible barcode label. Swab specimens were refrigerated (2–8 °C) upon arrival in the laboratory and were only removed during processing. For processing, swab specimen tubes were placed in racks and vortexed at 2200 rpm for 5–10 s using a Fisherbrand™ Microplate Standard Vortex Mixer (Thermo Fisher Scientific, Waltham, MA, USA), then allowed to sit for 5–15 min at room temperature (RT).

Saliva specimens were kept at RT or refrigerated (2–8 °C) until processing and then refrigerated until disposal. Saliva specimens were subjected to centrifugation at 1855× *g* and 25 °C for 5 min. If the saliva volume was at least 1.5 mL, it was transferred using the Biomek i5; if the volume was less than 1.5 mL, it was transferred manually and tested individually. 

**Figure 2:**
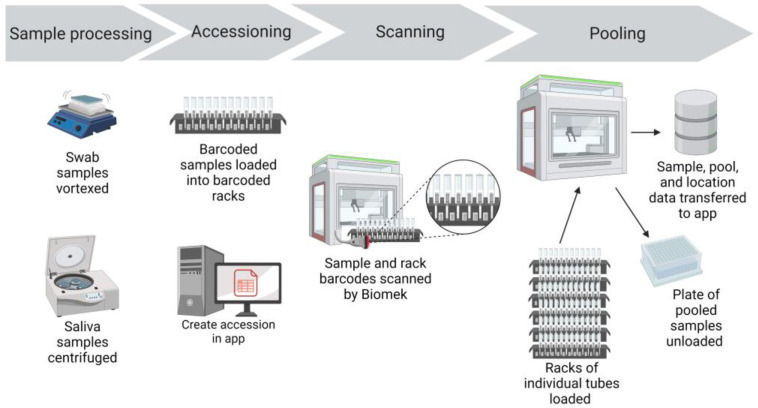
CCTL sample pooling workflow. After processing, specimen tubes were placed in Biomek tube racks so that the specimen barcode was visible between the rack position barcodes. A barcode scanner read specimen and rack position barcodes as the racks were loaded. The Biomek i5 generated pools of 5 specimens in a 96-deep-well plate. Specimen barcodes, pool constituents, and corresponding storage locations were captured and transferred to the COVID-19 receiving app. Figure created with BioRender.com, last accessed 5 June 2023.

Prepared specimen tubes were placed in Biomek tube racks so that the specimen barcode was visible between the rack position barcodes (Figure 2).

### 2.4. Specimen Accessioning, Pooling, and Transfer with the Biomek i5 Workstation

Specimens were transferred from 5 mL collection tubes to 96-deep-well plates for nucleic acid extraction using Biomek i5 automated workstations with high-efficiency particulate air (HEPA) filters (Beckman Coulter Life Sciences, Indianapolis, IN, USA). Biomek software version 5.1, Biomek Method Launcher version 3.2, and Data Acquisition and Reporting Tool (DART) 2.0 software were provided with the workstation (Beckman Coulter Life Sciences). The Biomek i5 automated workstations were operated inside benchtop biocontainment enclosures (bioBUBBLE model PU-1818-01-03, Fort Collins, CO, USA) for an additional layer of biosafety.

Once the specimens were ready to be scanned and pooled, the Create Accession Application (app) was opened. The appropriate submission source, specimen collection date, specimen type, and number of total specimens for the run were entered. The app automatically created the accession and records for each sample in the LIMS database. The sample identifications were initially created as an auto-incrementing number in the LIMS. The app also printed a set of barcoded labels with the run’s accession number. One accession label was placed on the 96-deep-well sample plate and the rest of the accession labels accompanied the specimen plate through the workflow. The labeled 96-deep-well sample plate was placed on the Biomek deck in preparation for sample transfer and pooling.

From the Biomek launcher software, the appropriate method was selected for the run (pooling or individual sample transfer, swabs, or saliva). The methods scripts were custom-designed using Python scripts and consisted of step-based workflows used by the Biomek liquid handler to process the samples. The first step was to scan the barcode label on the 96-deep-well sample plate, identifying the accession number that was created at the beginning of the workflow. Initiating and running the Biomek method step launched the Python script, which used the scanned barcode to connect to the LIMS database and determine the number of samples to be pooled or transferred. The Python script then updated the DART reports generated using Biomek. The reports specified the number of samples to be processed and which samples would be pooled into each well of the 96-deep-well plate. A full plate had 465 samples, pooled in groups of 5 samples per well (185 µL of each sample), thus creating 93 pools. If there were fewer than 465 samples to be scanned and tested that could not be evenly distributed into pools of 5, the final pool would have either 2, 3, or 4 samples. If the final pool was determined to have just one sample, then the script directed that the penultimate pool would have four samples and the final pool would have two samples. During the validation of this assay, the limit of detection (LOD) was determined to be 250 genome copies per mL and the diagnostic sensitivity for a pool of 5 samples was 93% [15].

Next, Biomek tube racks with specimen tubes were loaded into the Biomek. The Biomek tube racks also had position barcode labels that identified the location of each tube (Figure 2). As the racks were loaded into the Biomek, a barcode reader scanned the barcode of the rack position and the barcode of the specimen tube (Figure 2). These data were captured in the Biomek system to record the identity of each individual specimen and the location of that specimen.

The Biomek i5s used in our workflow were equipped with a Span-8 pipette head that could transfer 8 samples at a time into a 96-deep-well plate. An aliquot (185 μL) of each sample was dispensed into a single well, starting in column 1 and continuing for the remaining columns of the 96-deep-well plate. Pools were generated by repeating this process four additional times, until five samples were added to each well. The individual residual specimens remained separate and were stored refrigerated in case retesting or pool deconvolution was needed. On each 96-deep-well plate, 3 wells in column 12 were left empty to be used for negative (F12 and G12) and positive (H12) controls. Pooling 465 samples using the Biomek i5 and the script programmed to run the instrument took approximately 45 min.

Alternatively, if individual samples were transferred without pooling, up to 93 samples (200 μL each, accomplished with two transfers of 100 μL) were transferred into a 96-deep-well plate containing Proteinase K. The direct transfer of 93 individual samples with the Biomek i5 took approximately 15 min.

After the sample transfer was complete, sample plates were sealed with an adhesive film. Sealed plates were enclosed in a secondary container with a latched lid, along with the additional accession labels, and then the container was wiped with a disinfectant towel. The sample plate was then moved into the nucleic acid extraction room.

### 2.5. Specimen Identification and Pool Constituent Tracking

As the Biomek transferred samples into pools in the 96-deep-well plates, the DART software tracked which samples were pooled and recorded the position of each sample and pool in the plate. When the sample transfer was complete, two reports were automatically generated as part of the Biomek method. One file contained the barcode identity of the original specimen, the rack, and the rack position it was in. The second file contained the listing of the barcodes in each pool. The Biomek method launcher automatically saved these files to a secured network file server. Rhapsody monitored this network directory and automatically processed the files and updated the accession record in the LIMS database. The sample identification field in the LIMS was updated with the barcode ID from the specimen tube, and the pool number was saved as part of the sample record. The rack and position information were combined and saved as the storage location for the sample. The order and position of each specimen tube were maintained when they were transferred from the Biomek racks to storage racks identified with the respective accession labels. Each Biomek rack has 16 tube positions and storage racks were arrayed in 6 columns of 16 tubes so that the sample order and positions mirrored the order in which they were scanned and pooled. This allowed us to use the rack and position values to uniquely locate any sample for pool deconvolution or the retesting of individual samples.

As new accessions were created, they appeared in the working accessions tab of the application hub to provide real-time workflow tracking. Accession details and status were also displayed.

### 2.6. Automated Generation of Sample Plate Map Using the PCR App

The location of each sample in the 96-deep-well plate was maintained throughout the workflow so that pools could be traced back to the sample pool plate for confirmatory testing or deconvolution. From the PCR app, the appropriate accession number was entered, and the pool numbers or sample identities obtained from the barcode scanner populated a 96-well plate map without the need for manual data entry. Manual editing was optional so that individual samples or pools could be added to the plate. Once the map layout was complete, it was printed, and the hard copy accompanied the 96-deep-well plate throughout the laboratory workflow (Figure S1: example plate map for SARS-CoV-2 testing). The printed map had a designated space for an accession label and documented the identity of equipment used, the reagent lot numbers used, the initials of the technician completing each task, and the result analysis steps as part of the quality system implemented in the laboratory.

The PCR app also generated an electronic plate map to be uploaded into the ABI 7500 Fast real-time PCR instrument software so that samples and runs were labeled rapidly without manual entry, and the appropriate fluorescent detectors were selected.

### 2.7. Nucleic Acid Extraction

**Figure 3:**
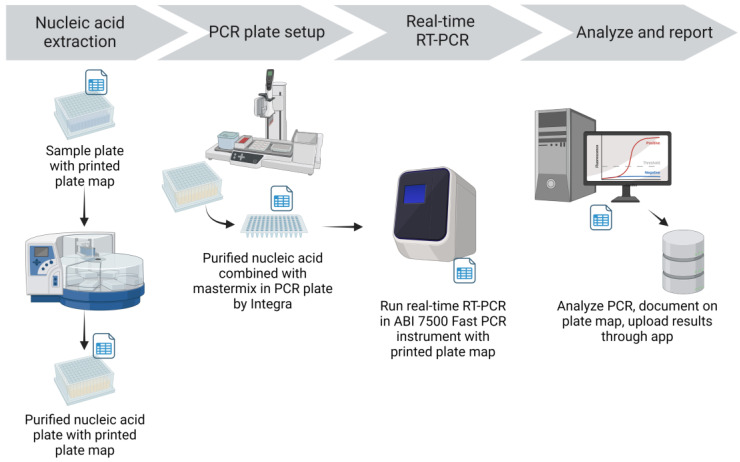
CCTL real-time PCR workflow. Nucleic acid was extracted using the KingFisher Flex and was added to the RT-PCR mastermix with an Integra pipetting robot. RT-PCR amplification and detection occurred on the ABI 7500 Fast platform. After analysis, results were uploaded to the COVID-19 PCR app for reporting. Figure created with BioRender.com, last accessed 5 June 2023.

Nucleic acid (NA) was extracted from specimens using the Applied Biosystems MagMax™ Viral/Pathogen II Nucleic Acid Isolation Kit and the KingFisher Flex Magnetic Particle Processor (Thermo Fisher Scientific Inc., Waltham, MA, USA) (Figure 3). Extraction reagents were aliquoted into 96-deep-well plates in batches in a dedicated area away from the sample processing and extraction areas. Integra Assist Plus pipetting robots (Integra Biosciences Corp., Hudson, NH, USA) with 12 channels were used to automate reagent transfer. Eighty percent ethanol for the Wash 2 plate was prepared daily and aliquoted into deep well plates by Integra Assist Plus pipetting robots. Wash 2 (80% ethanol) plates were labeled with the time and date of preparation to ensure they were used within 24 h. Prepared extraction reagent plates were sealed and stored in plastic containers with latched lids. The required amount of binding solution for the number of samples plus 18% overage (to provide sufficient volume for Integra transfer) was aliquoted into 50 mL tubes. As needed, the respective volume of beads was added to the binding solution and mixed by inversion. The tube containing the binding solution and beads was labeled with the time and date to ensure they were used within 24 h. The lot number of the extraction reagents was recorded on a printed extraction reagent recipe sheet and on the PCR plate map.

Nucleic acid extraction was performed in a dedicated extraction room that was isolated from the sample processing, RT-PCR mastermix preparation, and RT-PCR setup. Working in a biosafety cabinet with appropriate personal protective equipment, pools in the pooled sample plate were mixed 3 times by an Integra pipetting robot using filtered tips, and an aliquot of 200 μL was transferred into a 96-deep-well plate containing Proteinase K. For plates of individual sample tests, the Biomek i5 transferred samples directly into a deep-well plate containing Proteinase K. An aliquot of 200 μL VTM was added to the negative extraction control well (F12) on each plate. The prepared beads and binding solution were then added to the plate containing 200 μL sample and Proteinase K by the Integra pipetting robot. An accession label was added to the elution plate before extraction to maintain sample identity in the RT-PCR setup room. Sample and reagent plates were then loaded on the KingFisher Flex magnetic particle processor for automated NA extraction. Extraction was performed following manufacturer-provided scripts for the MagMax Viral/Pathogen II extraction kit, and the process took about 22 min. After extraction, the elution plate was sealed and placed on ice and then transferred to the RT-PCR setup room. The lot number of the extraction reagents used, the KingFisher Flex instrument identification, and the technician’s initials were recorded on the printed plate map.

### 2.8. SARS-CoV-2 RT-PCR Assay

The Tetracore EZ-SARS-CoV-2 RT-PCR assay (Tetracore, Inc., Rockville, MD, USA) was the assay of choice for testing [15] and was performed on ABI 7500 Fast real-time PCR instruments (Thermo Fisher Scientific Inc.) with Sequence Detection Software version 1.5.1. Briefly, the EZ-SARS-CoV-2 assay is a multiplex reaction including FAM-labeled probes targeting the N gene of SARS-CoV-2, a Cy5-labeled probe targeting the human RNase P gene, and a TAMRA-labeled probe targeting an inhibition control (IC).

RT-PCR mastermix was prepared as directed by the manufacturer in a dedicated clean room. If multiple plates were expected, larger batches of mastermix were prepared and aliquoted into RT-PCR plates with a dedicated Integra pipetting robot. Plates containing mastermix were covered, marked with the time of mastermix preparation to ensure use within 2 h, and placed in ice buckets in the RT-PCR setup room. The lot number of the EZ-SARS-CoV-2 reagents used, the date, the time, and the technician’s initials were recorded on the printed mastermix recipe sheet and on the PCR plate map.

In the dedicated RT-PCR setup room, elution plates were placed on the Integra pipetting robot base unit with the 96-well magnet installed to minimize the potential transfer of any beads remaining in the elution. PCR plates filled with mastermix were also placed on the Integra base, in the same orientation as the elution plate. The Integra pipetting robot was used to transfer the NA elutions into the RT-PCR plate containing mastermix, and the combined contents were mixed by pipetting up and down three times (Figure 3). Notations were made on the PCR plate map for any wells for which the appropriate volume of elution was not transferred; this was often due to elutions with high viscosity. If the appropriate elution volume could not be transferred with the Integra or a single-channel pipettor, the samples in that pool were tested individually. Assay controls (negative amplification control and positive amplification control (PAC) provided with the assay) were then added to the appropriate wells of the RT-PCR plate with a single channel pipettor. After sealing (PX1 PCR Plate Sealer, Bio-Rad, Hercules, CA, USA), the plates were centrifuged (plate microcentrifuge model C2000, Benchmark Scientific, Sayreville, NJ, USA) briefly to collect the contents at the bottom of the well. A visual inspection was performed to ensure equal volumes were present across all reaction wells. Using the ABI 7500 Sequence Detection software, the electronic plate map was imported, and the run was saved to the local drive. The filename of the run included the accession number(s), the date, the technician’s initials, and the real-time instrument identity. The RT-PCR plate was loaded into the ABI 7500 Fast real-time PCR instrument; the RT-PCR program took about 1 h and 26 min. The lot number of the EZ-SARS-CoV-2 reagents used, and the technician’s initials were recorded on the PCR plate map.

### 2.9. Result Analysis and Quality Control

Amplification conditions and analyses were performed as directed by the EZ-SARS-CoV-2 assay manufacturer. All amplification curves were inspected during analysis. Additionally, negative and positive control wells were assessed for expected outcomes. If the results of the control wells were not as expected, the assay was repeated. Amplification of the inhibition control was expected to result in a narrow range of Ct values within a run. If the inhibition control was not detected, the sample was determined to be invalid. If the human RNase P gene amplification signal resulted in a Cy5 Ct value of 37.05 or greater, the sample was determined to be invalid. This Cy5 Ct value cutoff was established from the amplification of serial dilution replicates, which revealed that Cy5 Ct values greater than 37.05 exhibited diminished repeatability. A cutoff was calculated for the SARS-CoV-2 target based on the FAM Ct values at the EZ-SARS-CoV-2 assay’s limit of detection; if the FAM Ct value of an individual sample was greater than 37.09, the test was repeated. If the second result was discordant (i.e., not detected), the result was determined to be inconclusive. Analysis and quality control checks were recorded on the PCR plate map, along with descriptions of any samples requiring additional review or repeated testing.

Once the analysis was completed, the results were exported from the sequence detection software to be reviewed using the upload results from the PCR app. Highlighting was used to distinguish the control results from the samples and to flag positive results or Ct values that exceeded cutoffs. Results that were ready to be finalized were uploaded to the LIMS. Results of “not detected” were automatically finalized and submitted via a secure file transfer protocol (SFTP) service in Amazon Web Services (AWS) to the CMC reporting system.

### 2.10. Deconvolution of Positive Pools

**Figure 4:**
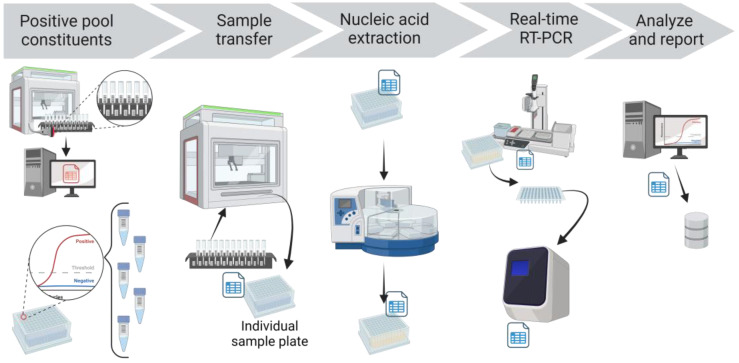
CCTL pool deconvolution workflow. Positive pool constituents and respective storage locations were obtained from the COVID-19 app. Individual samples were transferred by the Biomek i5, and a deconvolution plate map was generated by the app. The same extraction, PCR, analysis, and reporting workflow described above were followed. Figure created with BioRender.com, last accessed 5 June 2023.

If a SARS-CoV-2 FAM Ct value was detected for a pool, each constituent sample of that pool was tested individually (Figure 4). The PCR app automatically finalized the pool test code, but this initial detected result was not reported. Rather, the deconvolution test code was automatically added to the individual samples that comprised the pool. The individual samples and the respective storage locations were obtained from the Working CPOOL tab in the application hub, drawing from the information stored in the LIMS after the Biomek transfer. These individual samples were then recovered from the storage racks, plated using individual sample transfer protocols in Biomek, extracted, amplified, and analyzed with the same protocols. When the analyzed results of the individual deconvoluted samples were reviewed in the app and uploaded to the LIMS, they were automatically reported as the final result.

### 2.11. Daily Report

At the conclusion of testing each day, laboratory management used the daily reporting app to generate a PDF document summarizing all results finalized on that day. The results were broken down by accession, submission source, and results. Daily reports also included cumulative totals of tests, specimen types, and results. The daily report was automatically emailed to the laboratory management team and other designated individuals in the infection control group at the university.

### 2.12. Equipment and Instrumentation

The equipment and instrumentation used in the CCTL are listed in Table 1. The workflow in the laboratory was arranged in three testing lanes comprising three Biomek i5s, feeding four KingFisher Flex automated extractors and 10 ABI 7500 FAST real-time PCR instruments. This allowed sufficient redundancy that enabled the continued testing of the samples even in times when certain instruments were out of order.

### 2.13. Information Technology and Applications

A new restricted equipment network was created to isolate CCTL data and traffic. Access to this network was controlled through an access control list.

The Amazon Web Services (AWS, https://aws.amazon.com/) cloud platform was used to create and manage the server and application infrastructure.

The Oracle SE2 version 12.1.0.2.v17 (https://www.oracle.com/) database underlying the VetView (version 0.9.6C, http://vetview.org/) LIMS used by the Cornell Animal Health Diagnostic Center was duplicated to create a separate and dedicated instance of the LIMS for CCTL. The Oracle database ran in the AWS Relational Database Service, which met our requirements for high availability and durability.

Python (https://www.python.org/) scripts were used to automate the Biomek method and workflow. Specifically, Python scripts were also written to direct the Biomek pooling strategy based on the number of specimens in the run and to track the specimens included in each pool. Additionally, scripts were written to track specimen identification and storage locations recorded through the Biomek barcode scanner.

Rhapsody Integration Engine software version 6.2.2 (Lyniate Headquarters, Boston MA, USA) was used to (1) monitor the database for new DART report outputs from the Biomek and use that to update the accession record in VetView, (2) upload the result files to the secure FTP service in the AWS cloud platform, and (3) distribute the daily report.

**Figure 5:**
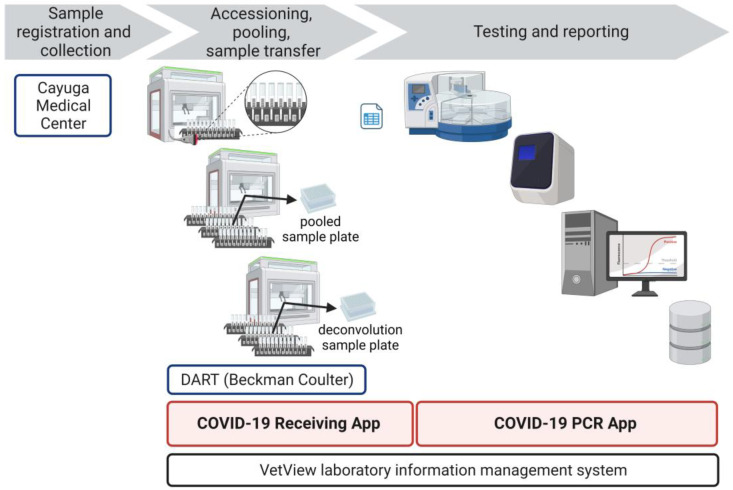
CCTL custom applications. Sample registration and collection were managed by Cayuga Medical Center. The COVID-19 receiving app captured sample barcode numbers, pool constituents, sample locations, and created accessions. The COVID-19 PCR app generated printed and electronic plate maps, analysis, and testing information. The app also interpreted uploaded results and reported them to Cayuga Medical Center. Figure created with BioRender.com, last accessed 5 June 2023.

Custom applications were built using Angular.js (https://angularjs.org/) framework for the front-end and Node.js (https://nodejs.org/en/) for the API back-end. The COVID-19 application hub was developed to provide a streamlined set of software apps to automate data entry and minimize manual input (Table 2, Figure 5).

Custom interfaces were either created or expanded by the Cayuga Health Integration Team to receive registrations, order requests, and test results via the Health Level 7 (HL7) message format from VetView LIMS and import them into Cayuga Medical Center’s electronic health record system, Meditech Expanse (Medical Information Technology, Inc., Canton, MA, USA). The dashboards were monitored using Microsoft Power BI. An existing reporting interface was used to report the results to the Electronic Clinical Laboratory Reporting System (ECLRS), the New York state reportable disease database.

SAP Crystal Reports software version 11.5.10 (https://www.sapstore.com/solutions/99043/SAP-Crystal-Reports?url_id=ctabutton-UnifiedSearchResult) was used to generate a daily report.

### 2.14. Statistical Analysis

Testing capacity, Ct values, and turnaround time data were compiled and analyzed using Microsoft Excel Office Professional Plus 2019. Plots were generated using GraphPad Prism version 9.1.2 for Windows, GraphPad Software, San Diego, CA, USA.

The results presented herein are based on SARS-CoV-2 testing from 17 August 2020 to 27 February 2023 unless noted otherwise. The first day of testing at CCTL was 17 August 2020 and 28 February 2023 marked the closure of the laboratory.

## 3. Results

### 3.1. SARS-CoV-2 Testing Capacity

**Figure 6:**
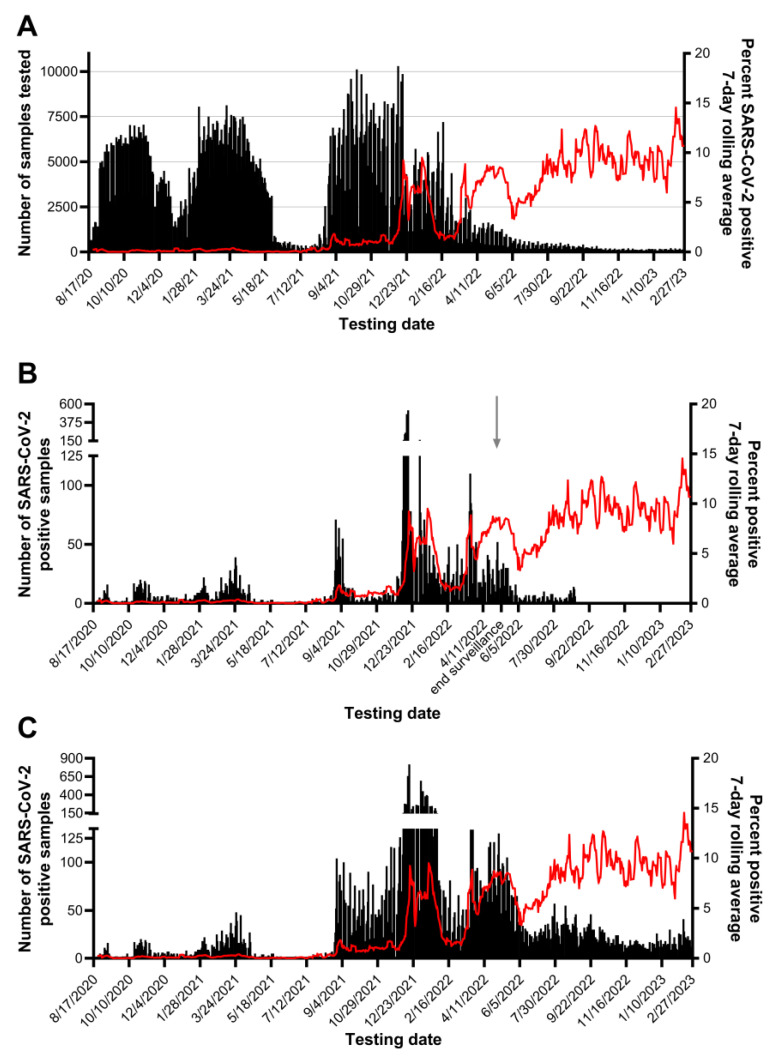
Number of samples tested and SARS-CoV-2 case counts and prevalence detected by CCTL. The date is presented on the x-axis. (**A**) The number of samples tested per day from 17 August 2020 to 27 February 2023 is on the left y-axis and SARS-CoV-2 prevalence calculated as the 7-day rolling average is shown by the red line on the right y-axis; (**B**) number of SARS-CoV-2-positive samples from the Cornell University population detected per day by CCTL from 17 August 2020 to 31 August 2022 when on-campus collection sites closed. The number of SARS-CoV-2-positive samples is on the left y-axis and the SARS-CoV-2 prevalence 7-day rolling average is shown by the red line on the right y-axis. Note the break in the left y-axis. The end of mandatory surveillance participation on 10 May 2022 is noted by an arrow on the x-axis; (**C**) number of SARS-CoV-2-positive samples submitted from the Cornell University population and surrounding community, detected per day by CCTL from 17 August 2020 to 27 February 2023. The number of SARS-CoV-2-positive samples is on the left y-axis and the SARS-CoV-2 prevalence 7-day rolling average is shown by the red line on the right y-axis. Note the break in the left y-axis.

In the fall of 2020, CCTL was equipped with two Biomek i5 automated workstations, two KingFisher Flex magnetic particle processors, and six ABI 7500 Fast real-time PCR instruments, which enabled the testing of up to 7058 samples per day and 38,447 samples per week (Figure 6). More equipment was obtained before the 2021 spring semester, including a third Biomek i5 automated workstation, two KingFisher Flex magnetic particle processors, and four additional ABI 7500 Fast real-time PCR instruments. With the hiring of additional staff, this increased capacity allowed the testing of up to 8127 samples per day and 42,441 samples per week in the spring of 2021 (Figure 6). The highest number of samples tested and reported in one day was 10,303 in December 2021. Surveillance testing was scheduled to be heaviest on weekdays and reduced on weekends. The testing demand decreased between the semesters and as students left campus at the end of the spring semester. Mandatory participation in the Cornell University SARS-CoV-2 surveillance program ended on 10 May 2022 and testing remained available on-campus until the collection sites closed on 31 August 2022. CCTL continued operations after 31 August 2022 to test the samples submitted to CMC.

### 3.2. Pooled Sample Performance

Between 17 August 2020 and 20 April 2022, the last day pooling was used; 370,839 pools were tested for SARS-CoV-2. The vast majority (369,949, 99.8%) of these pools were comprised of 5 samples. Some pools (890, 0.2%) contained four or fewer individual samples. A total of 12,347 SARS-CoV-2-positive pools were deconvoluted, and another 489 pools were deconvoluted because of quality control checks. Of the SARS-CoV-2-positive pools, 9968 (80.7%) identified one positive individual sample per pool, and 1430 (11.6%) pools contained two positive individuals. During the fall of 2021, when the prevalence of positive samples was high, pools with more positive individual samples were detected: 118 pools contained three positive individuals, 6 pools contained four positive individuals, and 1 pool contained five positive individuals. The number of SARS-CoV-2-positive individuals identified from the university population during this time frame is shown in Figure 6B. Notably, 5676 positive samples were identified from the university and the surrounding community during the month of December 2021 alone, accounting for 20.7% of all positive samples detected in the laboratory. The week of 13 to 19 December 2021 alone yielded 2478 positive samples, which corresponds to 9.1% of all positive results. Pooling was no longer used after 20 April 2022 because of the small number of samples submitted and the continued positivity rate of 5–10%.

SARS-CoV-2-positive pools with Ct values less than 37.09 (LOD of the EZ-SARS-CoV-2 RT-PCR assay [15]) successfully identified positive individuals 98.4% (10,913 of 11,095) of the time. When SARS-CoV-2 Ct values were greater than 37.09, a positive individual was identified in 55.9% (700 of 1252) of pools and no positive individuals were identified in 44.1% (552 of 1252). Out of the 370,839 tested pools, 734 (0.2%) yielded a positive pool result but a constituent individual positive was not identified. The majority (552 of 734, 75.2%) of these pools with discordant results had a SARS-CoV-2 Ct value of 37.09 or greater.

**Figure 7:**
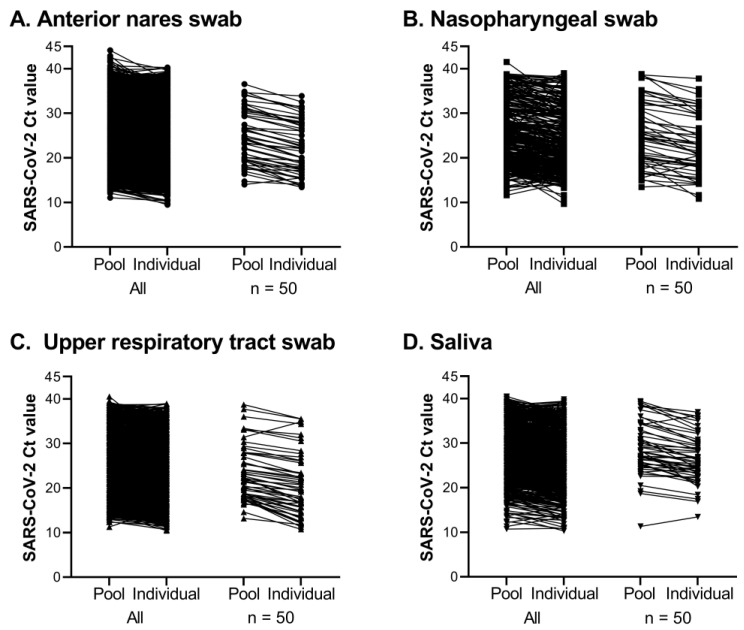
Comparison of SARS-CoV-2 Ct values between pooled and constituent positive individual samples for tests completed between 17 August 2020 and 31 December 2021. Lines connect Ct values of pools with their respective constituent positive sample. All pools of 5 with 1 positive are shown on the left for each specimen type; a subset of 50 results were randomly selected for the plot on the right.

The SARS-CoV-2 Ct values of positive pools ranged from 9.73 to 44.12. The expected difference in Ct values between a positive pool and one constituent positive sample, spanning a five-fold dilution, is 2.32. The average difference observed between the SARS-CoV-2 Ct value of a positive pool and the Ct value of the deconvoluted individual positive sample was 2.18 with a standard deviation of 1.82, calculated from 9925 pools that contained one positive individual out of five samples (Table 3, Figure 7). To maintain efficiency in the workflow, it was decided that AN and NP swab specimens could be pooled in the same run. When this occurred, the specimen type was entered as URT, to denote upper respiratory tract swabs (Table 3, Figure 7).

### 3.3. Turnaround Time

**Figure 8:**
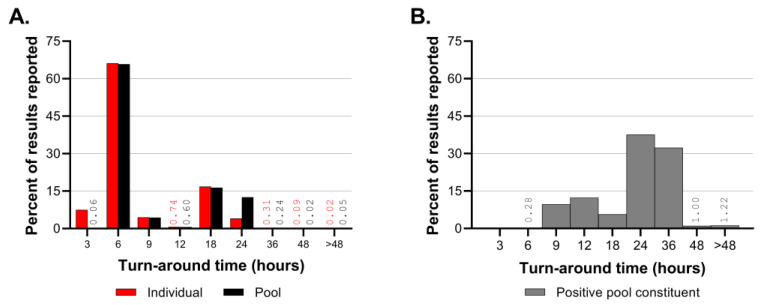
Turnaround time for SARS-CoV-2 tests, based on 2,079,635 results from 17 August 2020 to 27 February 2023. The turnaround time is binned by hours along the x-axis and the percent of results reported in that bin is shown on the y-axis. (**A**) Turnaround time for individual and pool results are shown in panel A; (**B**) turnaround times for positive pool constituent results are shown in panel B. Values are labeled when bars are not visible, with font color coordinating with test type.

The turnaround time (TAT) from sample accessioning to result reporting to the CMC system was evaluated for the duration of the CCTL operation (17 August 2020 to 27 February 2023). The turnaround time was calculated separately for pooled samples tested for surveillance purposes, samples tested individually to deconvolute pools, and samples tested individually for contact tracing, adaptive testing, or cause. The median turnaround time for the results of the pooled samples was 4 h and 46 min (Table 4), with a minimum TAT under 3 h. Although the maximum TAT for pooled samples was 51.5 h, only 0.3% of the pooled results had a turnaround time of over 24 h (Figure 8). The median TAT for the deconvolution of positive pools was 22.3 h (Table 4). The fastest TAT for pool deconvolution was 4 h and 12 min, while the longest deconvolution result was 72 h; the latter was due to an accession being pooled on Friday and deconvoluted on the following Monday. For samples tested individually, the median TAT was just under 4 h, approximately forty-five minutes less than the median TAT for pooled samples (Table 4). Individual samples could be resulted in just under 2 h; however, the results for one accession of individual samples took up to 72 h because they spanned a holiday weekend in a time when the lab was no longer operated 7 days a week.

Overall, the majority of results were reported between 3 and 6 h for the pooled and individual samples, whereas deconvolution results were most often reported between 24 and 36 h (Figure 8). Prior to September of 2021, 61.8% of deconvolution tests were reported between 6 and 12 h.

### 3.4. Assay and Laboratory Performance Monitoring

The monitoring of assay performance was enabled by capturing all results in the Internal Control Tracking Dashboard of the COVID-19 application hub. Each month, a review was executed detailing the number of reactions performed and summarizing the performance of sample and control reactions. The numbers of samples reported as invalid, inconclusive, unsuitable, or not tested were listed, and a brief explanation for the results was included.

The performance of the positive amplification control was plotted for the month to observe variability. Each positive amplification control Ct value observed above the expected pre-determined range was reviewed. Additionally, we evaluated whether an association existed between elevated positive amplification control Ct values and the ABI 7500 Fast real-time PCR instruments used during the month. The results from the negative extraction and negative amplification controls were reviewed for all runs every month, particularly confirming that no SARS-CoV-2 was detected in a negative control.

The performance of the inhibition control was evaluated each month by plotting all control fluorophore Ct values. Minor fluctuations in Ct values are expected between the plates; in our experience, a Ct value of approximately 26 was most commonly observed. A representative plot of the control fluorophore Ct values for the month of March 2021 (45,123 reactions) is provided in Figure S2.

Seven quality indicator goals were set as measures of laboratory and testing performance. The goals were: (1) to maintain a specimen submission rejection rate of less than 1%; (2) to report 95% of results within 24–48 h of sample receipt; (3) to maintain a rate of less than 5% corrected reports; (4) to have zero non-conformances; (5) for complaint investigations to be 90% completed within 2 weeks; (6) for staff to achieve proficiency test performance of 100% for each testing event; and (7) for 100% of staff competency assessments to be completed on time. Each month, these indicators were assessed to determine whether the stated goals were met. If a goal was not met, a root cause analysis was performed to identify corrective actions. In most cases, all the quality indicators were met on a regular basis demonstrating the robustness of the workflow established in the CCTL.

## 4. Discussion

The robust high-throughput laboratory workflow described above enabled us to meet Cornell University surveillance testing needs during the COVID-19 pandemic (with a maximum of 10,303 samples per day and 42,441 samples per week). The laboratory tested a total of 2,079,685 samples between 17 August 2020 and 27 February 2023. This capacity and the rapid turnaround time were made possible by automating several steps of our workflow, including specimen accessioning, pooling, nucleic acid extraction, and PCR set-up. The CCTL’s capacity exceeded the university’s surveillance needs and thus the lab performed overflow testing of community specimens in partnership with CMC.

The customized applications developed to support the workflow improved efficiency and minimized errors. We found it essential to involve application developers who understood the IT needs of a diagnostic laboratory and sample pooling workflow early in the process of establishing the laboratory. This enabled the rapid development of customized apps that enhanced the workflow. Continuous collaboration with the app developers allowed real-time improvements and updates in the apps as the workflow or the surveillance/testing programs changed. The specimen storage racking and tracking system devised specifically for the laboratory workflow was essential for efficiently accessing constituent specimens of positive pools for individual testing. The workflow also benefitted from the availability of the AWS cloud platform supporting the server and custom COVID-19 application infrastructure. Some IT solutions developed in CCTL are now being implemented in the Animal Health Diagnostic Center to support animal diagnostic testing and the performance of testing for the National Animal Health Laboratory Network (NAHLN).

Testing pools of five samples was essential to our workflow and seemed to be of the appropriate size to maximize efficiency without losing sensitivity. Of the 370,839 pools tested, SARS-CoV-2 was detected in 12,347 pools, and 94.1% were confirmed to contain one or more positive individual samples. Most positive pools that did not identify a positive individual had a high SARS-CoV-2 Ct value, with 75.2% at or beyond the assay’s limit of detection (Ct > 37.09). Good agreement was observed for the difference in Ct values between a positive pool and the constituent positive individual samples, regardless of specimen type. Requiring testing of all members of the community, the rapid identification of positive individuals and placement in isolation combined with rigorous contact tracing efforts minimized the spread of SARS-CoV-2 on campus and through the surrounding community during fall 2020 and spring 2021 semesters, as shown in Figure 6. The quick resolution of positive clusters emphasized the effectiveness of Cornell University’s surveillance program and the efficiency of this workflow. These observations also highlight the contribution of CCTL as one of the pillars of the successful control of SARS-CoV-2 infections at Cornell University.

The turnaround time was monitored regularly throughout CCTL’s operations. Turnaround time calculations began at accession creation rather than the sample collection time because the lab’s operation schedule fluctuated to adapt to the surveillance program’s needs. At the beginning of semesters when many people returned to the Ithaca, NY, area and when the SARS-CoV-2 prevalence of positive samples was high, a same-day result turnaround was expected. However, workflow efficiency was hampered by the rate of sample collection and delivery when performing same-day testing. When the SARS-CoV-2 prevalence was low, the lab shifted to next-day testing so that all samples that would be tested were delivered the previous evening. When sample numbers were low, a hybrid system was employed in which samples from the previous day were tested as well as samples collected early on the same day. During fall 2021, the lab was closed on Saturdays, and, occasionally, samples delivered to the laboratory late on Friday night were pooled, but testing and reporting were not finalized until Sunday afternoon, resulting in maximum pool test turnaround times of 51 h and positive pool deconvolution turnaround times of 72 h.

Staffing the laboratory through fluctuating testing volumes and SARS-CoV-2 prevalence was challenging. In the fall of 2020, the lab operated with fewer than 10 staff, largely drawing on existing Animal Health Diagnostic Center staff experienced in molecular diagnostic testing. In preparation for higher testing demands, six more full-time technicians were hired for the Spring 2021 semester. When the sample volume and the prevalence of positive samples were low over the summer of 2021, many staff returned to or were hired by the Animal Health Diagnostic Center. Most of those staff were recalled to CCTL for student arrival testing in late August. From August 2020 to December 2021, CCTL was operated in one and a half shifts 5 days a week (Mon–Fri) and half shifts on Saturdays and Sundays. The ability to flex the number of staff assigned to the CCTL was instrumental in our success.

Within two years of the detection of the index SARS-CoV-2 case in Wuhan, China, in December 2019 [1], five genetic variants, designated variants of concern (VOCs) by the World Health Organization (WHO), emerged and sequentially replaced the preceding strain [16,17]. These VOCs prompted renewed waves of infections and disease, which increased diagnostic testing demands. Of particular significance to Cornell University and the surrounding Tompkins County area was the arrival of the Delta VOC during summer 2021 and Omicron in December 2021. The Delta (B.1.617.2) COVID-19 variant was reported in Tompkins County on 10 August 2021 and accounted for 92% of the positive samples collected during June and July of 2021 and later sequenced in a collaboration with the Cornell University Virology Laboratory at the Animal Health Diagnostic Center [18]. The prevalence of SARS-CoV-2-positive samples was low during the summer of 2021, with a 7-day rolling average below 0.4%. We observed an increase in prevalence up to 1.8% during student arrival testing in late August 2021 driven by the Delta variant, and then a plateau between 0.67 and 1.2% through the fall months.

Detection of the Omicron (B.1.1.529) COVID-19 variant was first reported by Tompkins County on 11 December 2021 via the sequencing of positive cases performed at the Cornell University Virology Lab, (https://tompkinscountyny.gov/health/COVID-19-omicron-variant, last accessed on 5 June 2023). The following day yielded the largest increase in cases on a single day since the onset of the pandemic in Tompkins County (https://tompkinscountyny.gov/health/COVID-19-increase-cases-121221, last accessed on 5 June 2023). In the following week, 2478 positive samples were reported by our laboratory; this represents 22.7% of all positive results determined by the laboratory up to that point. The Omicron variant spread rapidly through the Cornell University and Tompkins County population, reaching a peak incidence of 9.21% on a single day, despite the fact that more than 70% of this population was fully vaccinated. Omicron was initially described in South Africa and classified as a VOC on 26 November 2021 by the World Health Organization, shortly before its detection in Ithaca, NY [17,19]. Both the speed of Omicron transmission and the susceptibility of fully vaccinated individuals observed in the Tompkins County area were consistent with Omicron epidemiological characteristics reported globally [20,21,22,23].

The emergence of the Delta and Omicron VOCs and the resultant increase in the prevalence of positive samples affected the laboratory’s workflow leading to longer turnaround times for pool deconvolution. Throughout the first 16 months of SARS-CoV-2 testing, the majority of pooled samples and individual test results were reported within 3–6 h. The TAT for positive pool constituent testing shifted from predominantly 6–12 h prior to September 2021 to predominantly 18–36 h in the fall of 2021. The number of samples requiring positive pool constituent testing increased from nearly 1200 to nearly 3800 in the month of September and exceeded 20,000 in December 2021. Although the Delta and Omicron outbreaks had a significant impact on the CCTL workflow and turnaround time due to the magnitude of the outbreaks and the number of positive samples, the robust workflow established at CCTL was still capable of handling the increased volume and testing needs through these outbreaks. It is important to note, however, that these two major efforts were supported by the availability of trained staff from the Animal Health Diagnostic Center which were transferred into CCTL to provide relief and increased testing capacity.

The high-throughput diagnostic RT-PCR workflow described above was designed for low-prevalence scenarios but had built-in flexibility and redundancy to accommodate expansion in emergency outbreaks. The increase in the prevalence of positive samples during December 2021 had a significant impact on the workflow, increasing the number of staff and hours needed to complete the work and extending the usual TAT. Unfortunately, the spike in prevalence coincided with two of three Biomek i5 workstations being out of service. The usage of the remaining Biomek i5 workstation was alternated between pooling incoming samples and the individual testing of positive pool constituents. If a high-prevalence scenario is anticipated, the workflow may need additional equipment dedicated to positive pool constituent testing. It is difficult to set a prevalence threshold to define when additional equipment and staff are needed because the workflow is affected by the number of incoming samples and the prevalence. For example, the highest number of positive results identified in one day was 816 positive samples out of 9862 tested on 17 December 2021; this necessitated positive pool constituent testing to be performed on 3415 samples, which accounted for 65% of the reactions performed and 34% of the results reported on that day. The incidence of that single day was 8.3%; although there have been days with higher incidence, the workflow was not negatively impacted because fewer total samples were submitted.

## 5. Conclusions

Overall, the high-throughput workflow established at CCTL performed very well for the intended purpose: intense surveillance testing of a large university population. Given its high efficiency, this high-throughput workflow could be modeled to establish regional One Health testing hubs for infectious disease preparedness and emergency responses. Currently, there are several animal and human health pathogens circulating in animal hosts or in humans worldwide in addition to SARS-CoV-2 such as highly pathogenic avian influenza virus (HPAI), swine influenza virus (IAV-S), Ebola virus (EBOV), Nipah virus (NiPV), porcine epidemic diarrhea virus (PEDV), and African swine fever virus (ASFV), which could result in large epidemics or pandemics in which high-throughput testing will be needed [24,25,26,27,28,29,30].

## Figures and Tables

**Table 1:** Cornell COVID-19 Testing Laboratory Equipment list.

Purpose	Equipment
Sample collection	Brady i7100 printers
Sample processing	Biosafety Cabinet The Baker Company SG403A
	Fisherbrand Microplate Standard Vortex Mixer
	Centifuge Jouan GT-4 22
Sample pooling/transfer	Beckman Coulter Biomek i5 Span8 Enclosed with HEPA filter
	Biomek racks
	bioBUBBLE Biobubble model PU-1818-01-03
Nucleic acid extraction	Thermo Fisher Scientific KingFisher Flex Magnetic Particle Processor model 5400630
Mastermix	Mystaire PCR workstation MY-PCR24
PCR setup	AirClean Systems AirClean 600 PCR workstation model AC648TLFUVC
	Corning Mini Microcentrifuge model 6770
	BioRad PX1 PCR Plate Sealer
	Benchmark Scientific Plate Microcentrifuge model C2000
PCR amplification and detection	ABI 7500 Fast Real-Time PCR System
Integra Automated Pipetting Systems	Integra Assist Plus pipetting robot base, Part number 4505
	Voyager 12 channel adjustable tip spacing electronic pipettors: 50 μL, 12.5 μL
	Viaflo 12 channel electronic pipettors: 1250 μL, 300 μL, 125 μL, 12.5 μL
Pipettors	Eppendorf Xplorer plus 12 channel pipettors: 50–1200 μL, 15–300 μL
	Eppendorf Xplorer plus 8 channel pipettors: 0.5–10 μL
	Rainin L12-10XLS+ 12 and 8 channel manual pipettors:10 μL
	Single channel manual pipettors 100–1000 μL, 20–200 μL, 2–20 μL, 1–10 μL
General equipment	Refrigerators (2–8 °C)
	Freezers (−20 °C, −80 °C)
	Ice machine

**Table 2:** COVID-19 Application Hub functions.

Task	App Name	Details and Customizable Actions
Create accession for pooled or individual samples	Receiving App	Specifies whether pooled or individual transfer, the number of specimens, specimen type, date collected, and submission source
Generate printable plate map for selected accession	PCR App	Can edit wells manually if needed Can add samples to empty wells manually
Create an electronic plate map to import to real-time PCR instrument	PCR App	Imported plate map includes sample id and appropriate detectors for selected assay (EZ-SARS-CoV-2)
Review results before reporting	PCR App	Lists all sample and control well Ct values and result interpretations Confirms instrument and plate identification are present Can change or hold result if needed
Display real-time status of accessions	Receiving App	Provides number of samples, number of pools, number of finalized results, and how many samples reported as detected for each accession
Display real-time status of pools being tested or samples being deconvoluted from pools	Receiving App	Provides individual sample identification with corresponding pool number and storage location
Generate daily report	Daily Reporting App	Obtain and email report of sample volume and results for specified day
**Lab performance monitoring:**		
View assay control performance	Internal Control Tracking Dashboard	Can limit data to selected control, run, date or range of dates, real-time PCR instrument, or detector
Turnaround time tracking	Business intelligence	Obtain turnaround time for a sample, day, or range of days
Daily totals	Business intelligence	View daily report for specified date
**Other tools:**		
Barcode lookup tool	Utility App	Find the corresponding accession number, submission source, specimen type, sample date, pool number, reported date, and result for a specified barcode
List samples with results other than not detected	Business intelligence	Searchable list of specimen details for those reported as detected, invalid, inconclusive, and unsuitable
View of number of labels printed at collection sites	N/A	Track sample collection at campus sites in real time
Printing or re-printing barcode labels	Receiving App	Specify specimen or accession label to be printed
Printer setup for authorized users	N/A	Designate printers for users

‘N/A’ indicates not applicable.

**Table 3:** Comparison of SARS-CoV-2 Ct values between pooled and constituent positive samples.

Specimen Type	Number of Positive Pools with One Positive Constituent	Average Ct Difference	Standard Deviation Ct Difference
Anterior nares swab	5135	2.11	1.53
Nasopharyngeal swab	344	2.04	2.68
Upper respiratory tract swab	2450	2.13	1.52
Saliva	1996	2.46	2.49
All specimen types	9925	2.18	1.82

**Table 4:** CCTL turnaround time sorted by type of SARS-CoV-2 test ^1^.

	Individual Test	Pool Test	Positive Pool Constituent Test
Number of results ^2^	227,579	1,788,415	63,641
Minimum turnaround time	1:53	2:40	4:12
Median turnaround time	3:55	4:46	22:18
Maximum turnaround time	72:25	51:35	72:04

^1^ Turnaround time displayed in hours:minutes format. ^2^ Number of results from 17 August 2020 to 27 February 2023.

## Data Availability

Custom scripts developed to support this workflow are available upon request.

## Supplementary Materials

The following supporting information can be downloaded at: https://www.mdpi.com/article/10.3390/v15071555/s1, Figure S1: Example sample plate map for SARS-CoV-2 testing, Figure S2: Plot of internal control Ct values for the month of March 2021.
