# Supplementary material for: The Cornell COVID-19 Testing Laboratory: A Model to High-Capacity Testing Hubs for Infectious Disease Emergency Response and Preparedness
Source: Viruses. 2023 Jul 15;15(7):1555. doi: 10.3390/v15071555 (PMC10385863; doi:10.3390/v15071555)
Supplement: Supplementary file 1 [file viruses-15-01555-s001.zip › viruses-2471211-supplementary.pptx]

## Slide 1
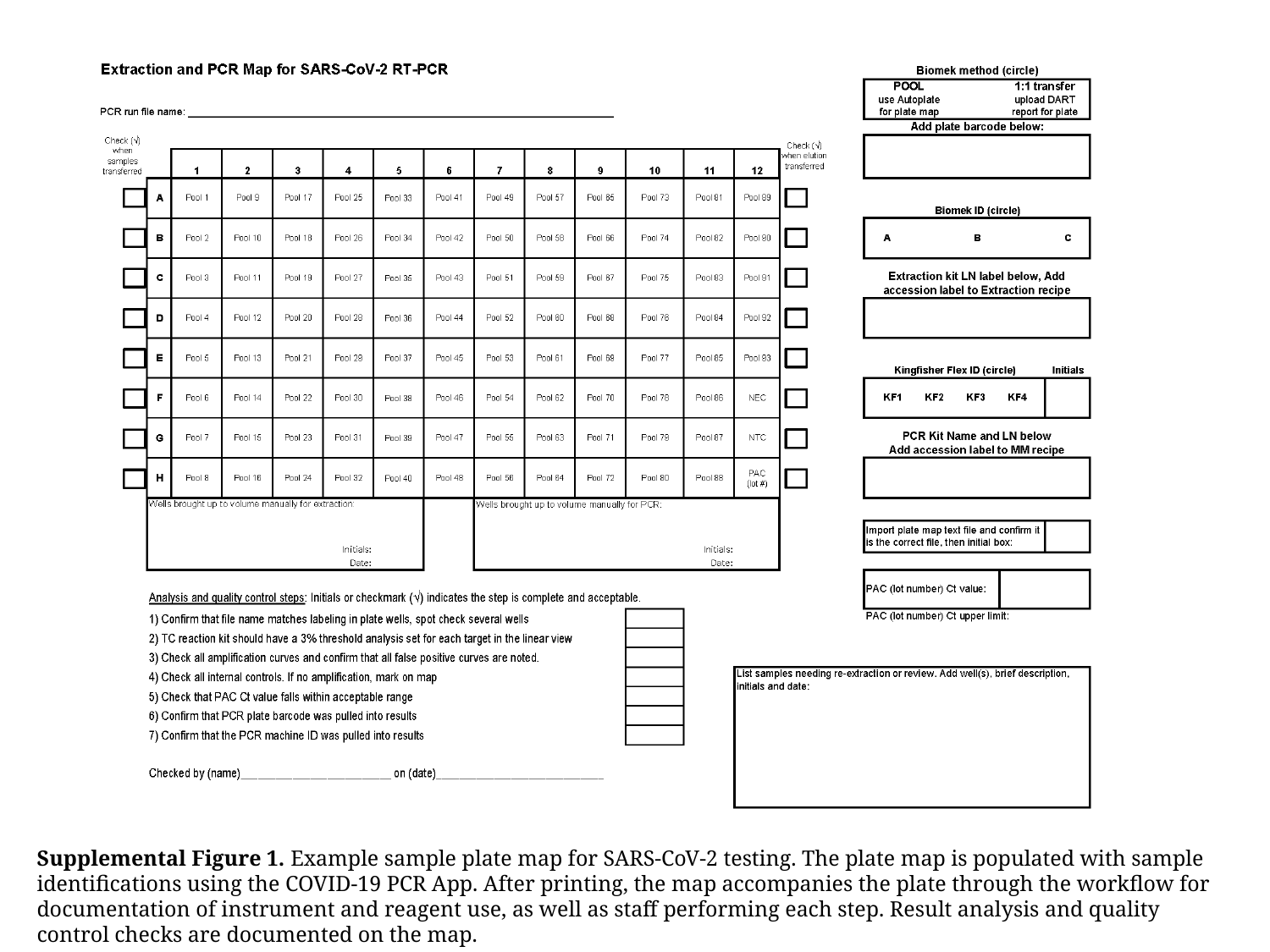

Supplemental Figure 1. Example sample plate map for SARS-CoV-2 testing. The plate map is populated with sample identifications using the COVID-19 PCR App. After printing, the map accompanies the plate through the workflow for documentation of instrument and reagent use, as well as staff performing each step. Result analysis and quality control checks are documented on the map.

## Slide 2
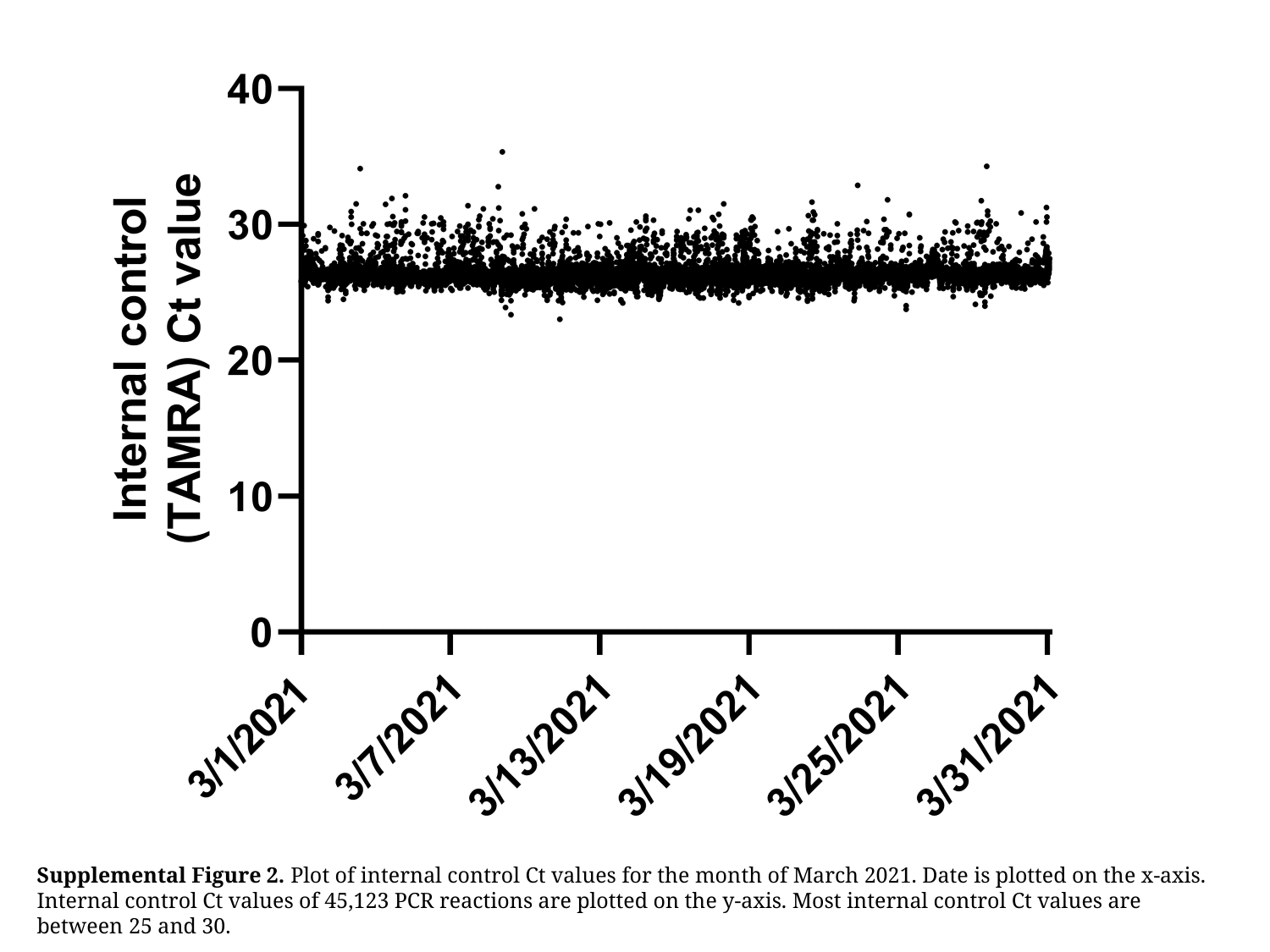

Supplemental Figure 2. Plot of internal control Ct values for the month of March 2021. Date is plotted on the x-axis. Internal control Ct values of 45,123 PCR reactions are plotted on the y-axis. Most internal control Ct values are between 25 and 30.
